# *Toona sinensis* Inhibits LPS-Induced Inflammation and Migration in Vascular Smooth Muscle Cells via Suppression of Reactive Oxygen Species and NF-**κ**B Signaling Pathway

**DOI:** 10.1155/2014/901315

**Published:** 2014-03-04

**Authors:** Hsin-Ling Yang, Pei-Jane Huang, Yi-Ru Liu, K. J. Senthil Kumar, Li-Sung Hsu, Te-Ling Lu, Yi-Chen Chia, Tokuko Takajo, Anzai Kazunori, You-Cheng Hseu

**Affiliations:** ^1^Institute of Nutrition, China Medical University, Taichung, Taiwan; ^2^Department of Health and Nutrition Biotechnology, Asia University, Taichung, Taiwan; ^3^Department of Cosmeceutics, College of Pharmacy, China Medical University, Taichung, Taiwan; ^4^Institute of Biochemistry and Biotechnology, Chung Shan Medical University, Taichung, Taiwan; ^5^School of Pharmacy, College of Pharmacy, China Medical University, Taichung, Taiwan; ^6^Department of Food Science and Technology, Ta-Jen University, Ping-Tung County, Taiwan; ^7^Department of Medico Pharmaceutical Science, Nihon Pharmaceutical University, Komuro, Inamachi, Kita-Adachigun,Saitama, Japan

## Abstract

*Toona sinensis* is one of the most popular vegetarian cuisines in Taiwan and it has been shown to possess antioxidant, antiangiogenic, and anticancer properties. In this study, we investigated the antiatherosclerotic potential of aqueous leaf extracts from *Toona sinensis* (TS; 25–100 *μ*g/mL) and its major bioactive compound, gallic acid (GA; 5 *μ*g/mL), in LPS-treated rat aortic smooth muscle (A7r5) cells. We found that pretreatment with noncytotoxic concentrations of TS and GA significantly inhibited inflammatory NO and PGE_2_ production by downregulating their precursors, iNOS and COX-2, respectively, in LPS-treated A7r5 cells. Furthermore, TS and GA inhibited LPS-induced intracellular ROS and their corresponding mediator, p47^phox^. Notably, TS and GA pretreatment significantly inhibited LPS-induced migration in transwell assays. Gelatin zymography and western blotting demonstrated that treatment with TS and GA suppressed the activity or expression of MMP-9, MMP-2, and t-PA. Additionally, TS and GA significantly inhibited LPS-induced VEGF, PDGF, and VCAM-1 expression. Further investigation revealed that the inhibition of iNOS/COX-2, MMPs, growth factors, and adhesion molecules was associated with the suppression of NF-**κ**B activation and MAPK (ERK1/2, JNK1/2, and p38) phosphorylation. Thus, *Toona sinensis* may be useful for the prevention of atherosclerosis.

## 1. Introduction

Inflammation is an important event in the development of vascular diseases, including hypertension, atherosclerosis, and restenosis. Additionally, the inflammatory response is widely accepted as an essential event for smooth muscle cell (SMC) activation and atherosclerosis [1]. In response to inflammatory stimuli, bacterial lipopolysaccharide (LPS), activates SMCs to induce the expression of proinflammatory mediators, including inducible nitric oxide synthase (iNOS) and cyclooxygenase-2 (COX-2), which are responsible for the induction of nitric oxide (NO) and prostaglandin E_2_ (PGE_2_), respectively, and are known to promote SMC injury and cardiovascular diseases [2, 3]. A key signaling pathway involved in inflammation is the nuclear factor-*κ*B (NF-*κ*B) pathway [4]. LPS can induce NF-*κ*B activity in several cell types, including aortic SMC [5]. NF-*κ*B has also been implicated in the regulation of numerous growth factors and adhesion molecules in vascular SMCs, including vascular endothelial growth factor (VEGF), platelet-derived growth factor (PDGF), and vascular cell adhesion molecule-1 (VCAM-1), which play key roles in the recruitment of leukocytes to sites of inflammation [5, 6]. Therefore, in the vascular endothelium, NF-*κ*B activation leads to the induction of proinflammatory cytokines, growth factors, adhesion molecules, and chemoattractant proteins that contribute to all aspects of inflammation and cardiac diseases [7].

Matrix metalloproteinases (MMPs) and plasminogen activator (PA), particularly MMP-2, MMP-9, and tissue-type PA (t-PA), induce the breakdown of the extracellular matrix and allow for the migration of aortic SMCs from the tunica media to the intima, resulting in neointima hyperplasia [8, 9]. A recent report by Li et al. demonstrated that exposing human aortic SMCs to bacterial LPS markedly increased the expression of MMP-9, and this induction was regulated by activation of the TLR4/NF-*κ*B signaling pathways [10]. Thus, targeting NF-*κ*B-mediated activation of inflammation and migration in aortic SMCs holds promise for the development of novel antiatherosclerotic therapies.

Overproduction of ROS has been implicated in many aspects of vascular injury and lesion development, including lipoprotein oxidation, foam cell formation, and SMC hypertrophy, and it also promotes migration and proliferation in vascular SMCs [11]. ROS and induced oxidative stress are considered to be general mechanisms for NF-*κ*B activation by a variety of agents [4]. In addition, ROS generation is associated with the activation of mitogen-activated protein kinases (MAPKs), including extracellular signal-regulated kinase1/2 (ERK1/2), c-JUN N-terminal kinase1/2 (JNK1/2), and p38 MAPK, which are key transducers of extracellular signals that promote cell growth and migration [12]. The migration of vascular SMCs plays a central role in the pathogenesis of atherosclerosis, restenosis, and vascular graft stenosis [13].

*Toona sinensis* Roem (Meliaceae; TS) is a type of arbor that is widely distributed in Asia. In Chinese and Taiwanese cultures, TS is one of the most popular vegetarian cuisines. It has long been used as a traditional Chinese medicine for a wide variety of conditions, including rheumatoid arthritis, cervicitis, urethritis, tympanitis, gastric ulcers, enteritis, dysentery, itchiness, and cancer [14]. While the underlying pharmacological mechanisms of TS remain unclear, various biological activities of TS leaf extracts have been reported. Recent scientific investigations demonstrated that aqueous leaf extracts of TS possess a variety of biological activities, including antioxidant, anticancer, anti-inflammation, antidiabetes, and antiangiogenesis effects, as well as the ability to inhibit Leydig cell steroidogenesis and improve the quality and dynamic activity of human sperm [15–18]. The safety levels and nontoxic characteristics of TS were evaluated using acute and subacute toxicity studies in mice and rats, and no lethal effects were found at concentrations as high as 1 g/kg body weight [19, 20]. Our previous study demonstrated that TS and gallic acid (GA) exhibited anti-inflammatory effects in LPS-induced macrophage cells and a mouse model [15]. However, the effect of TS and GA against LPS-induced vascular SMC inflammation and migration is poorly understood. Therefore, the aim of the present study was to investigate the antiatherosclerotic properties of TS and GA in LPS-activated rat aortic smooth muscle (A7r5) cells.

## 2. Materials and Methods

### 2.1. Reagents

Dulbecco's modified Eagle's medium (DMEM), fetal bovine serum (FBS), and penicillin/streptomycin were obtained from Gibco/BRL Life Technologies Inc. (Grand Island, NY, USA). Lipopolysaccharide (LPS), 3-(4,5-dimethylthiazol-2-yl)-2,5-diphenyltetrazolium bromide (MTT), and 2′,7′-dihydrofluorescein diacetate (DCFH_2_-DA) were purchased from Sigma-Aldrich (St. Louis, MO, USA). Antibodies against iNOS, COX-2, MMP-2, MMP-9, t-PA, VEGF, PDGF, VCAM-1, and *β*-actin were purchased from Santa Cruz (Heidelberg, Germany). Antibodies against I-*κ*B*α*, NF-*κ*B, phos-ERK1/2, ERK1/2, phos-JNK, JNK, phos-p38 MAPK, and p38 MAPK, were obtained from Cell Signaling Technology Inc. (Danvers, MA, USA). Anti-p47^phox^ antibody was purchased from Merk-Millipore (Darmstadt, Germany). All other chemicals were reagent or HPLC grade and supplied by either Merck (Darmstadt, Germany) or Sigma (St. Louis, MO, USA).

### 2.2. Preparation of Aqueous Leaf Extracts of TS

TS leaves were obtained from Fooyin University, Kaohsiung, Taiwan. A voucher specimen was characterized by Professor Horng-Liang Lay (Graduate Institute of Biotechnology, National Pingtung University of Science and Technology, Pingtung County, Taiwan) and deposited at Fooyin University (Kaohsiung, Taiwan). Aqueous leaf extracts of TS were prepared by adding 1000 mL of water to 1000 g of fresh TS leaves, and this mixture was boiled until 100 mL remained, as previously described [21, 22]. The crude extracts were centrifuged at 3000 ×g for 12 min and the supernatant was used for this study. The crude extracts (50 g) were concentrated in a vacuum and freeze dried to form powder, with the stock subsequently stored at −20°C for further analysis of its anticancer properties. The yield of TS extracts was 6%. The total phenolic content of the TS extracts was estimated to be 130 ± 26 mg gallic acid (pyrocatechol) equivalents/g of plant extracts as described previously [23].

### 2.3. Isolation of Gallic Acid from TS Extracts

TS extracts were dissolved in a mobile phase consisting of methanol-water (50 : 50, v/v) and then subjected to high-performance liquid chromatography (HPLC) analysis and separation. Chromatographic separation was achieved with a mobile phase consisting of methanol-water (50 : 50, v/v) in the first 15 min, gradually increasing the methanol concentration to 100% over the next 10 min. A flow rate of 4.0 mL/min at room temperature was used. Eight compounds (gallic acid, methyl gallate, ethyl gallate, kaempferol, kaempferol-3-*O*-*β*-D-glucoside, quercetin, quercitrin, quercetin-3-*O*-*β*-D-glucoside, and rutin) were isolated from TS extracts. To identify these compounds, their spectral data (IR, NMR, and mass) were compared with analogous information reported in the literature [23, 24]. Gallic acid (GA), a natural phenolic component purified from TS extracts, was the main subject of this study, and it was produced with a yield of 10% [16].

### 2.4. Cell Culture and Sample Treatment

A rat aortic smooth muscle cell line (A7r5) was obtained from the American Type Culture Collection (ATCC, Manassas, VA) and grown in DMEM medium supplemented with 10% fetal calf serum, 100 *μ*g/mL penicillin, and 1 *μ*g/mL streptomycin, at 37°C with 5% CO_2_ in humidified conditions. Cultures were harvested, and the cell number was determined by counting cell suspensions with a hemocytometer. Prior to starting an experiment, cells were starved overnight, TS (25–100 *μ*g/mL) or GA (5 *μ*g/mL) was added for 2 h, and the supernatant was removed. Then, the cells were washed with PBS, and the culture media was replaced with serum-free medium, with or without LPS (100 ng/mL), for the indicated time points. LPS was dissolved in phosphate-buffered saline (PBS) (137 mM NaCl, 1.4 mM KH_2_PO_4_, 4.3 mM Na_2_HPO_4_, 2.7 mM KCl, pH 7.2).

### 2.5. MTT Assay

The effect of TS and GA on A7r5 cell viability was monitored using the MTT colorimetric assay. In brief, 2 × 10^5^ cells/well were plated in 12-well plates, and they were pretreated with or without various concentrations of TS (25–100 *μ*g/mL) or GA (5 *μ*g/mL) in absence of LPS for 12 h or in the presence of LPS (100 ng/mL) for 24 h. After treatment, the cells were incubated with 400 *μ*L of 0.5 mg/mL MTT in PBS for 2 h. Culture supernatants were removed and resuspended in 400 *μ*L of isopropanol to dissolve the MTT formazan, and the absorbance was measured at 570 nm using an ELISA microplate reader. The effect of TS and GA on cell viability was assessed as the percent of viable cells compared to the vehicle-treated control group, which was arbitrarily assigned to represent 100% viability. This assay was performed in triplicate for each concentration.

### 2.6. Measurement of ROS Generation

Intracellular ROS accumulation was detected by fluorescence microscopy, using DCFH_2_-DA. A7r5 cells were plated at a density of 2 × 10^5^ cells/well in a 12-well plate and cultured in DMEM supplemented with 10% FBS. The culture medium was renewed when the cells reached 80% confluence. After TS (25–75 *μ*g/mL) and GA (5 *μ*g/mL) treatment for 2 h, the cells were treated with LPS for 5 min. Then, the cells were further incubated with 10 *μ*M DCFH_2_-DA in culture medium at 37°C for 30 min. The acetate groups on DCFH_2_-DA were removed by intracellular esterase, trapping the probe inside the A7r5 cells. Then, the cells were rinsed with warm PBS buffer. The production of ROS was measured as the changes in fluorescence due to the intracellular accumulation of DCF caused by the oxidation of DCFH_2_. The intensity of DCF fluorescence was measured with a fluorescence microscope (Olympus 1 × 71 at 200x magnification). Fluorescence intensity was quantified from a square section of fluorescently stained cells, using analysis LS 5.0 soft image solution (Olympus Imaging America Inc., Corporate Parkway Centre Valley, PA, USA), and the fold increase of fluorescence intensity (ROS generation) was calculated compared to vehicle-treated control cells.

### 2.7. Immunofluorescence Assay

A7r5 cells (1 × 10^4^ cells/well) were cultured in DMEM with 10% FBS in an 8-well Lab-Tek chamber (Thermo Fisher Scientific, Waltham, MA, USA). The cells were pretreated with various concentrations of TS (75 *μ*g/mL) or GA (5 *μ*g/mL) for 2 h and then incubated with or without LPS for 40 min. After treatment, the culture medium was removed, and the cells were washed with PBS, fixed in 2% paraformaldehyde for 15 min, and permeabilized with 0.1% Triton X-100 for 10 min. Then, the cells were blocked with 10% FBS in PBS and incubated for 2 h with a specific anti-p47^phox^ primary antibody in 1.5% FBS. A FITC (488 nm) secondary antibody was incubated for another 1 h in 6% BSA. The cell nuclei were stained with a 1 *μ*g/mL DAPI solution for 5 min. The stained cells were washed with PBS and visualized using a fluorescence microscope at 200x magnification.

### 2.8. Determination of Nitric Oxide Production

The concentration of NO in the culture supernatant was determined by measuring the accumulated nitrite (NO_2_^−^), a major stable product of NO, using the Griess reagent colorimetric assay. A7r5 cells (2 × 10^5^ cells/mL) were cultured in 12-well plates and pretreated with TS (25–100 *μ*g/mL) or GA (5 *μ*g/mL) for 2 h prior to LPS (100 ng/mL) stimulation for 8 h. The culture supernatant (100 *μ*L) was mixed with the same volume of Griess reagents, and the absorbance of the mixture was read at 540 nm using an ELISA microplate reader. A standard curve was constructed using known concentrations of sodium nitrite.

### 2.9. Determination of PGE_2_ Production

The PGE_2_ concentration in the culture media was determined using a PGE_2_ ELISA kit (R and D Systems, Minneapolis, MN, USA). Briefly, A7r5 cells (2 × 10^5^ cells/mL) were cultured in 12-well plates and incubated with TS (25–100 *μ*g/mL) or GA (5 *μ*g/mL) for 2 h prior to the addition of LPS (100 ng/mL) for 8 h. The culture supernatant (100 *μ*L) was collected, and the PGE_2_ concentration was determined using an ELISA microplate reader, according to the manufacturer's protocol.

### 2.10. Protein Isolation and Western Blot Analysis

A7r5 cells (2 × 10^6^ cells) were seeded in a 10 cm dish. Once the cells reached 80% confluence, they were incubated with or without the indicated concentrations of TS or GA for 2 h. Then, these cells were stimulated by addition of LPS (100 ng/mL) for 5 min–12 h. The treated cells were washed once in cold PBS and suspended in 100 *μ*L of lysis buffer (10 mM Tris-HCl (pH 8), 0.32 M sucrose, 1% Triton X-100, 5 mM EDTA, 2 mM DTT, and 1 mM phenylmethyl sulfonyl fluoride). The suspension was vortexed, kept on ice for 20 min, and then centrifuged at 15000 ×g for 20 min at 4°C. Total protein content was quantified using the Bio-Rad protein assay reagent (Bio-Rad, Hercules, CA, USA), with bovine serum albumin (BSA) as the standard. The protein extracts were reconstituted in sample buffer (0.062 M Tris-HCl, 2% SDS, 10% glycerol, and 5% *β*-mercaptoethanol), and the mixture was boiled at 94°C for 5 min. Equal amounts (50 *μ*g) of the denatured proteins were loaded into each lane, separated by electrophoresis on 8%–15% SDS polyacrylamide gels, and the proteins were transferred to PVDF membranes overnight. The membranes were blocked with 0.1% Tween-20 in Tris-buffered saline containing 5% nonfat dry milk for 20 min at room temperature and then incubated with the indicated primary antibody for 2 h. The membranes were then incubated with a horseradish peroxidase-conjugated goat anti-rabbit or anti-mouse antibody for 2 h and developed using a chemiluminescence substrate (Millipore, Billerica, MA, USA). For densitometric analysis, band intensities were quantified using commercially available software (AlpaEaseFc 4.0; Genetic Technologies, Inc., Miami, FL, USA).

### 2.11. Cell Migration Assay

Migration assays were performed using transwell chambers. Briefly, 2 × 10^6^ cells/dish were seeded in a 10 cm dish overnight in DMEM supplemented with 10% FBS. Then, the medium was replaced with serum-free DMEM for 12 h, and the cells were treated with or without TS (25–75 *μ*g/mL) and GA (5 *μ*g/mL) for 2 h in 1% FBS medium. The cells were collected and centrifuged at 500 ×g for 3 min. The treated cells were seeded at a density of 1 × 10^5^ cells/well in 1% FBS medium in the upper chamber and challenged with LPS (100 ng/mL). The lower chamber was filled with complete medium. The cells were allowed to migrate for 24 h at 37°C. After the incubation, the cells that did not migrate, which remained on the top surface of the membrane, were removed with a cotton swab. The cell that migrated to the bottom side of the membrane were fixed in cold 75% methanol for 15 min and washed three times with PBS. Next, the cells were stained with Giemsa stain solution and then destained with PBS. Images were obtained using an optical microscope (200x magnification), and invading cells were quantified by manual counting. The percent inhibition of invading cells was quantified, with untreated (control) cells representing 100%.

### 2.12. Gelatin Zymography Assay

The amount of MMP-2, MMP-9, and t-PA in the cell culture medium was measured by gelatin zymography assay. A7r5 cells (3 × 10^5^ cells/well) were seeded into 12-well culture plates and grown until nearly confluent in complete medium. The cells were resuspended in 1% FBS medium and preincubated with various concentrations of TS (25–75 *μ*g/mL) and 5 *μ*g/mL of GA for 2 h prior to LPS (100 ng/mL) stimulation for 24 h. After treatment, the culture medium was collected and total protein was quantified using Bio-Rad protein assay reagents. An equal amount of culture samples, prepared without boiling or reduction, were mixed with 1 mg/mL gelatin or 50 *μ*g/mL of plasminogen and subjected to 8% SDS-PAGE electrophoresis. After electrophoresis, the gels were washed with 2.5% Triton X-100 and incubated in the developing buffer (50 mM Tris-base, 200 mM NaCl, 5 mM CaCl_2_, and 0.02% Brij 35) at 37°C for 24 h. Then, the gels were stained with Coomassie brilliant blue R-250. Clear bands on the blue background represent areas of gelatinolysis. The digestion of these bands was quantified using a gel documentation system (Fujifilm LAS-3000 imager), and densitometric analyses of band intensities were quantified using commercially available software (AlpaEaseFc 4.0).

### 2.13. NF-*κ*B Luciferase Reporter Assay

A7r5 cells (4 × 10^5^ cells/well) were cultured in a 6-well plate. After overnight incubation, the cells were cotransfected with pNF-*κ*B-SEAP and the pIRES-hrGFP-1a expression vector (10 : 1) using Lipofectamine 2000 (Invitrogen Corp., Carlsbad, CA, USA). Six hours after transfection, the cells were resuspended in serum-free medium, preincubated with or without various concentrations of TS (25–75 *μ*g/mL) or GA (5 *μ*g/mL) for 2 h, and stimulated by LPS (100 ng/mL) for 3–12 h. SEAP activity in the medium was evaluated using the Phospha-Light system, according to the manufacturer's protocol (Applied Biosystems, Bedford, MA, USA). Relative SEAP activity was determined to reflect the transcriptional activity of NF-*κ*B, and it was expressed as the fold increase relative to the luciferase intensity of untreated controls.

### 2.14. Statistical Analysis

The results are presented as the means and standard deviations (mean ± SD). All data were analyzed using analysis of variance (ANOVA), followed by Dunnett's test for pairwise comparison. All the experiments were performed in triplicate, and the statistical significance was defined as *P* < 0.05 for all tests.

## 3. Results

### 3.1. Effects of TS and GA on Cell Viability in LPS-Induced A7r5 Cells

**Figure 1:**
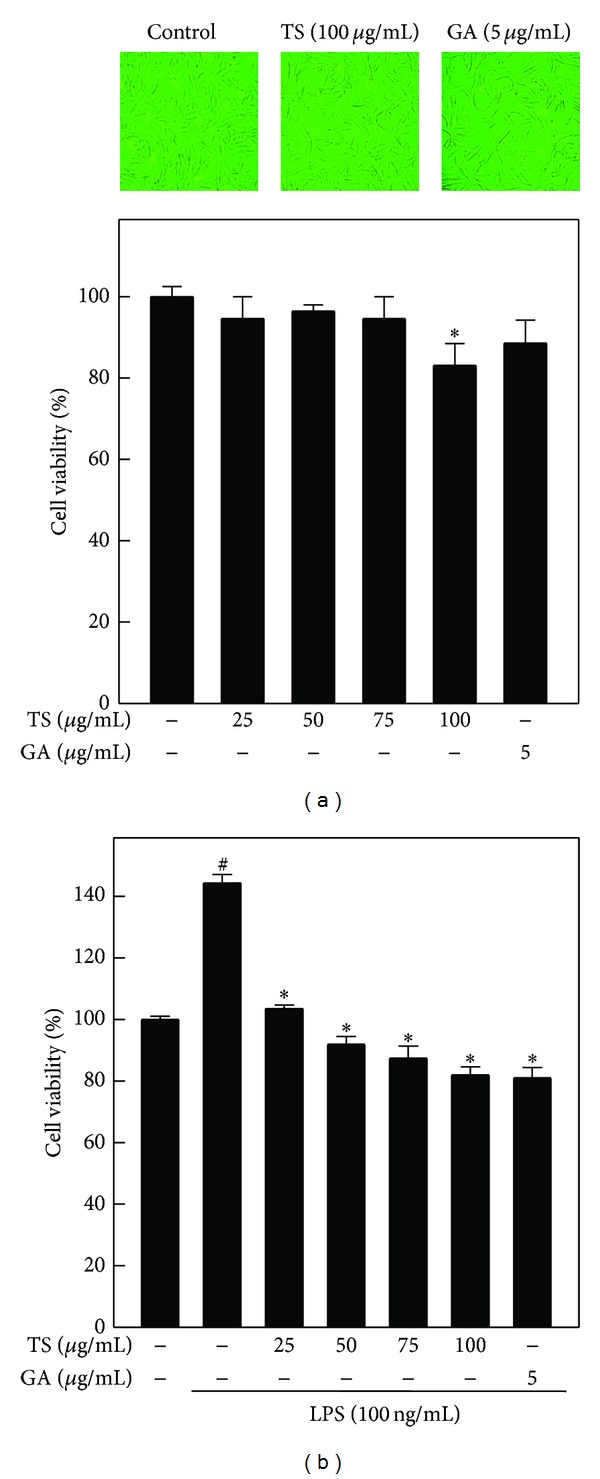
Effect of TS and GA on A7r5 cell viability. (a) Rat aortic SMC (A7r5) cells were incubated with TS (0, 25, 50, 75, and 100 *μ*g/mL) and GA (5 *μ*g/mL) for 12 h. (b) A7r5 cells were pretreated with TS (0, 25, 50, 75, and 100 *μ*g/mL) and GA (5 *μ*g/mL) for 2 h and then stimulated by LPS (100 ng/mL) for 24 h. The cell cultures were harvested, and cell viability was determined using the MTT assay (200x magnification). The percentage (%) of cell viability was calculated by the following equation: (A_570_ of treated cells/A_570_ of untreated cells) × 100. The results represent the mean ± SD of three independent assays. ^#, ∗^indicates significant difference (*P* < 0.05) compared to the control or LPS-treated groups, respectively. TS, *Toona sinensis*; GA, gallic acid.

To determine effective treatment concentrations, the cytotoxic effects of TS and GA were examined using the MTT colorimetric assay, in the presence or absence of LPS.  Figure 1(a) shows the percentage of viable cells after treatment with various concentrations of TS (25–100 *μ*g/mL) and a single concentration of GA (5 *μ*g/mL) for 12 h. The MTT assays showed that TS was not cytotoxic to A7r5 cells at concentrations up to 75 *μ*g/mL, whereas cell viability was reduced to 89% in response to 100 *μ*g/mL of TS. Moreover, GA (5 *μ*g/mL) was not cytotoxic toward A7r5 cells over the time course examined (Figure 1(a)). A7r5 cell morphology was examined using an optical microscope (200x magnification), and no morphological changes were observed in response to TS (100 *μ*g/mL) or GA (5 *μ*g/mL) treatment (Figure 1(a)). To further examine whether TS and GA inhibit LPS-induced A7r5 cell proliferation, the relative number of cells was determined by the MTT assay.  Figure 1(b) shows that A7r5 cell proliferation was greatly increased by LPS (from 100% to 147%), whereas TS and GA pretreatment significantly (*P* < 0.05) inhibited LPS-induced proliferation (Figure 1(b)).

### 3.2. TS and GA Inhibit LPS-Induced ROS Generation in A7r5 Cells

**Figure 2:**
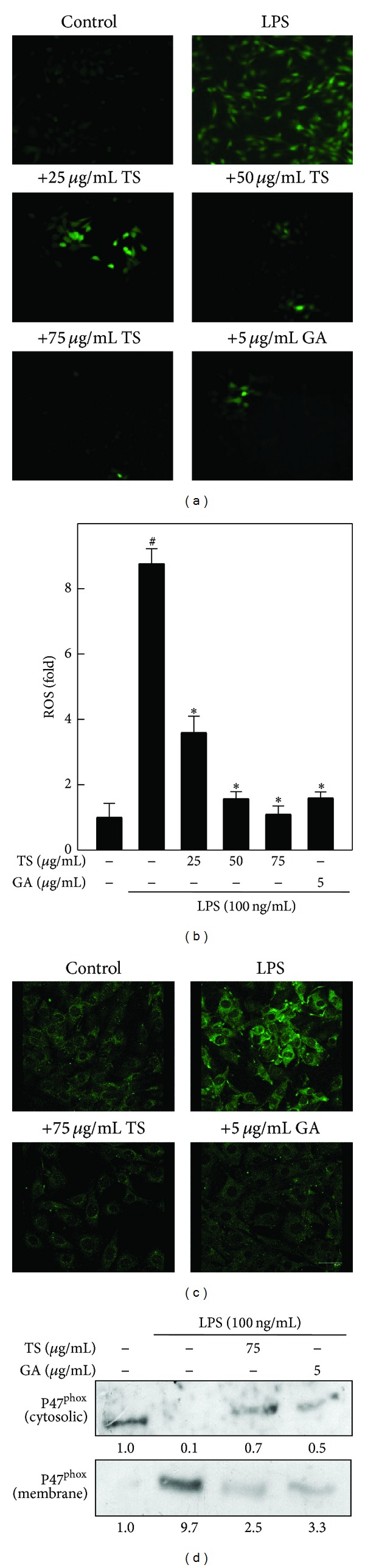
TS and GA inhibit LPS-induced ROS production and p47^phox^ expression in A7r5 cells. ((a), (b)) Cells were preincubated with or without TS (25–75 *μ*g/mL) or GA (5 *μ*g/mL) for 2 h and then stimulated with LPS (100 ng/mL) for 5 min. (a) Intracellular ROS levels were measured using a DCFH_2_-DA fluorescence microscope (200x magnification). The nonfluorescent cell membrane-permeable probe, DCFH_2_-DA, was added to the culture medium at a final concentration of 10 *μ*M. DCFH_2_ reacts with cellular ROS and is metabolized into fluorescent DCF. (b) The fluorescence intensity of DCF-stained cells was quantified as a percentage, using Olympus soft image solution software. ((c), (d)) TS and GA inhibited LPS-induced p47^phox^ membrane translocation in A7r5 cells. Cells were preincubated with or without TS (75 *μ*g/mL) or GA (5 *μ*g/mL) for 2 h and then stimulated by LPS (100 ng/mL) for 40 min. (c) The expression levels of p47^phox^ in treated A7r5 cells were determined by immunofluorescence, using a p47^phox^ specific primary antibody and a fluorescein isothiocyanate- (FITC-) conjugated secondary antibody (green). The subcellular distribution of p47^phox^ was photographed on a fluorescence microscope (400x magnification). (d) Cytoplasmic and membrane bound p47^phox^ protein expression was measured by western blot analysis using cytoplasmic and membrane fractions. Relative changes in protein bands were measured using AlpaEaseFc 4.0 software. Densitometric analysis, with the control being 100%, is shown just below the gel data. The results are presented as the mean ± SD of three independent experiments. ^#, ∗^indicates a significant difference (*P* < 0.05) compared to the control or LPS-treated groups, respectively.

Next, we used a DCFH_2_-DA fluorescent staining method to determine whether TS and GA were able to inhibit LPS-induced ROS generation in A7r5 cells. As shown in Figure 2(a), the intracellular ROS generation was dramatically increased in LPS-treated cells as indicated by increased intensity of DCF fluorescence, whereas this LPS-induced ROS generation was significantly inhibited by TS (25–75 *μ*g/mL) in a dose-dependent manner. GA-treatment (5 *μ*g/mL) resulted in a similar inhibitory effect on ROS generation. In addition, densitometric analyses show that exposing A7r5 cells to LPS led to a nearly 8.8-fold increase in ROS generation compared to the unchallenged control cells (1-fold). Importantly, pretreatment with TS significantly reduced the LPS-induced ROS generation (10-fold) to 3.6-fold, 1.6-fold, and 1.1 fold by 25, 50, and 75 *μ*g/mL, respectively (Figure 2(b)). GA also inhibited the LPS-induced ROS generation to 1.7-fold in A7r5 cells. These results imply that TS and GA may protect the vascular smooth muscle cells from LPS-induced oxidative stress by scavenging ROS and/or free radicals.

### 3.3. TS and GA Inhibit LPS-Induced p47^phox^ Membrane Translocation in A7r5 Cells

p47^phox^ is a catalytic subunit of NADPH oxidase that is mainly localized in the cytoplasm. Upon stimulation, p47^phox^ translocates to the membrane and induces superoxide generation. Since TS and GA inhibit LPS-induced ROS generation in A7r5 cells, we sought to determine whether TS and GA downregulate LPS-induced p47^phox^ activation and membrane translocation as well. Fluorescence microscopy revealed that in unstimulated cells, p47^phox^ was barely detectable in either the cytoplasm or membrane (Figure 2(c)). In contrast, after LPS treatment for 40 min, p47^phox^ expression increased, and it was predominantly clustered at the membrane. However, pretreatment with TS (75 *μ*g/mL) or GA (5 *μ*g/mL) significantly prevented LPS-induced p47^phox^ expression and membrane translocation in A7r5 cells (Figure 2(c)). Consistent with the results of fluorescence microscopy analyses, western blot analyses showed a significant increase in p47^phox^ expression in the membrane fraction after treatment with LPS for 40 min, and this increase in the membrane was significantly blocked by TS and GA pretreatment (Figure 2(d)). In addition, the amount of cytoplasmic p47^phox^ was directly proportional to the amount of p47^phox^ in the membrane in both the LPS and treatment groups. Therefore, TS and GA downregulate p47^phox^ expression to inhibit LPS-induced ROS production in A7r5 cells.

### 3.4. TS and GA Inhibit NO Production and iNOS Expression in A7r5 Cells

**Figure 3:**
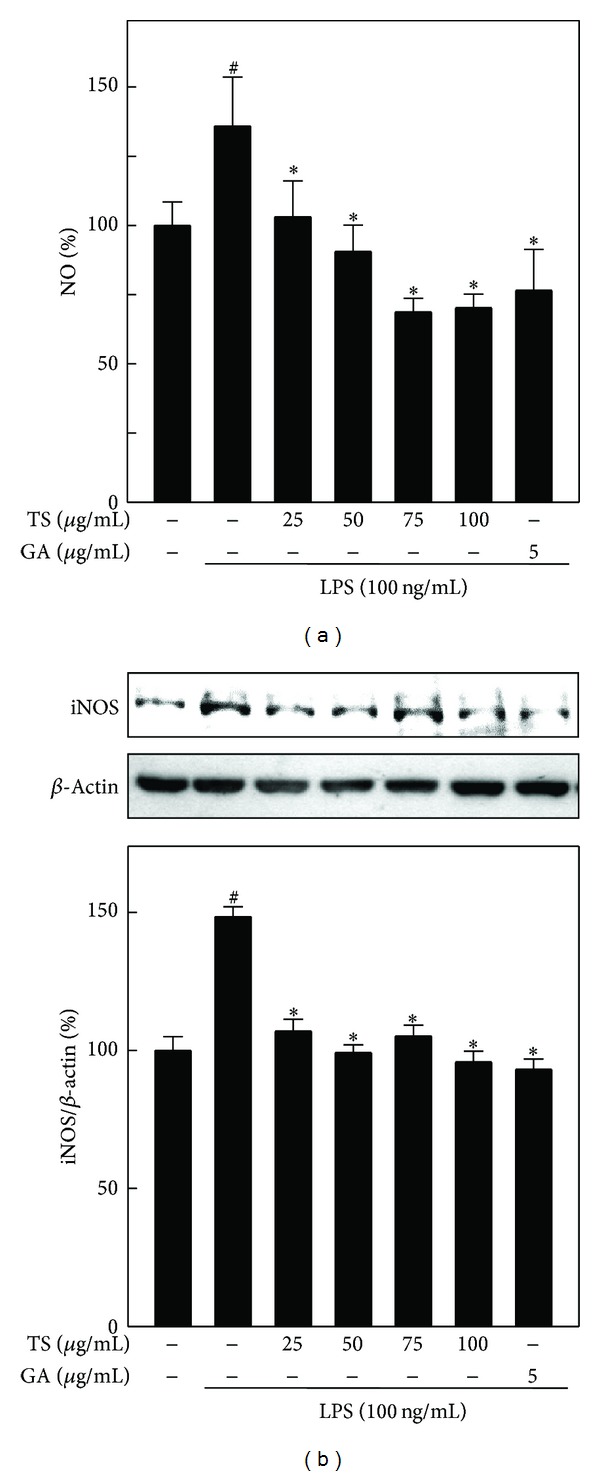
TS and GA inhibit NO production through the downregulation of iNOS protein expression in LPS-activated A7r5 cells. Cells were preincubated with or without TS (25–100 *μ*g/mL) or GA (5 *μ*g/mL) for 2 h and then stimulated with LPS (100 ng/mL) for 8 h. (a) NO production was determined by measuring the formation of nitrite, the stable end-metabolite of NO. (b) Protein (50 *μ*g) from each sample was resolved by 8% SDS-PAGE, and western blotting was performed. The results represent the mean ± SD of three assays. ^#, ∗^indicates significant difference (*P* < 0.05) in comparison to control or LPS-treated groups, respectively.

To investigate the anti-inflammatory effects of TS and GA, we first examined the inhibitory effects of TS and GA on LPS-induced NO production in A7r5 cells. A7r5 cells were preincubated with increasing concentrations of TS (25–100 *μ*g/mL) and GA (5 *μ*g/mL) and stimulated with LPS for 8 h. Extracellular (culture medium) NO levels were directly measured by quantifying its oxidized product, nitrite (NO_2_^−^). As shown in Figure 3(a), a significant (*P* < 0.05) increase in NO production was observed after exposure to LPS (136%), whereas pretreatment with TS and GA caused a sustained decrease in LPS-induced NO production. Furthermore, the TS- and GA-induced decrease in NO was comparatively lower than the basal level. Next, we hypothesized that the TS- and GA-induced inhibition of NO production was due to the downregulation of their catalytic enzyme, iNOS. As shown in Figure 3(b), iNOS protein expression was barely detectable in unstimulated control cells, whereas LPS treatment markedly (150%) increased iNOS expression in A7r5 cells. The increase in iNOS expression in response to LPS was inhibited by TS in a dose-dependent manner, and a similar inhibitory effect was also observed in response to GA treatment.

### 3.5. TS and GA Inhibit PGE_2_ Production and COX-2 Expression in A7r5 Cells

**Figure 4:**
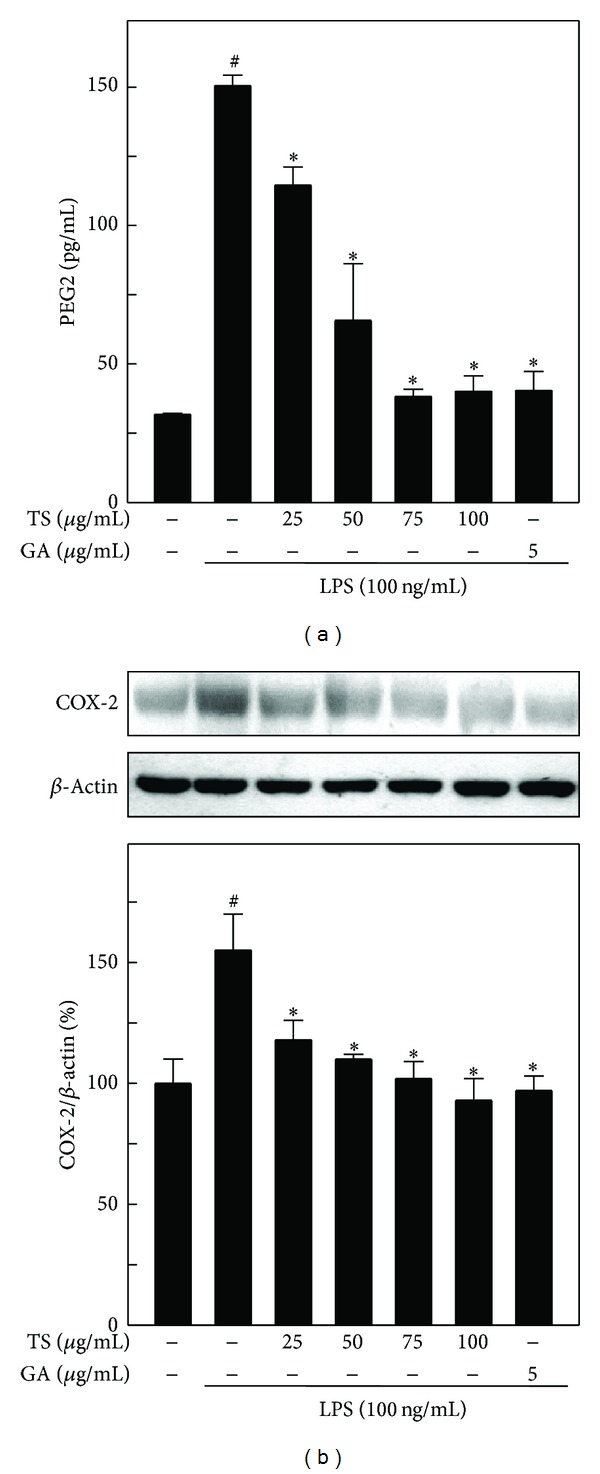
TS and GA inhibit PGE_2_ production through the downregulation of COX-2 protein expression in LPS-activated A7r5 cells. Cells were preincubated with or without TS (25–100 *μ*g/mL) or GA (5 *μ*g/mL) for 2 h and then stimulated with LPS (100 ng/mL) for 8 h. (a) PGE_2_ production was measured using a commercially available EIA kit, as described in the methods. (b) Protein (50 *μ*g) from each sample was resolved by 10% SDS-PAGE, followed by western blotting. The results represent the mean ± SD of three assays. ^#, ∗^indicates significant difference (*P* < 0.05) in comparison to control or LPS-treated groups, respectively.

During inflammation, inflammatory cells produce large amounts of PGE_2_, which is critically involved in vasodilation and pain at the inflammatory site. Therefore, we examined the effects of TS (25–100 *μ*g/mL) and GA (5 *μ*g/mL) on PGE_2_ production in LPS-induced A7r5 cells. As shown in Figure 4(a), after stimulating A7r5 cells with LPS for 8 h, PGE_2_ levels markedly increased to 150.5 pg/mL compared to the unstimulated control cells (31.7 pg/mL). However, pretreatment with TS or GA decreased PGE_2_ secretion in a dose-dependent manner (Figure 4(a)). Next, we hypothesized that the TS and GA-induced inhibition of PGE_2_ production may be due to the downregulation of their catalytic enzyme, COX-2. As shown in Figure 4(b), COX-2 protein expression was barely detectable in unstimulated control cells, whereas LPS treatment markedly (150%) increased COX-2 expression throughout the time course examined. However, both TS and GA inhibited the LPS-induced increase in COX-2 expression in a dose-dependent manner.

### 3.6. TS and GA Inhibits Migration of A7r5 Cells

**Figure 5:**
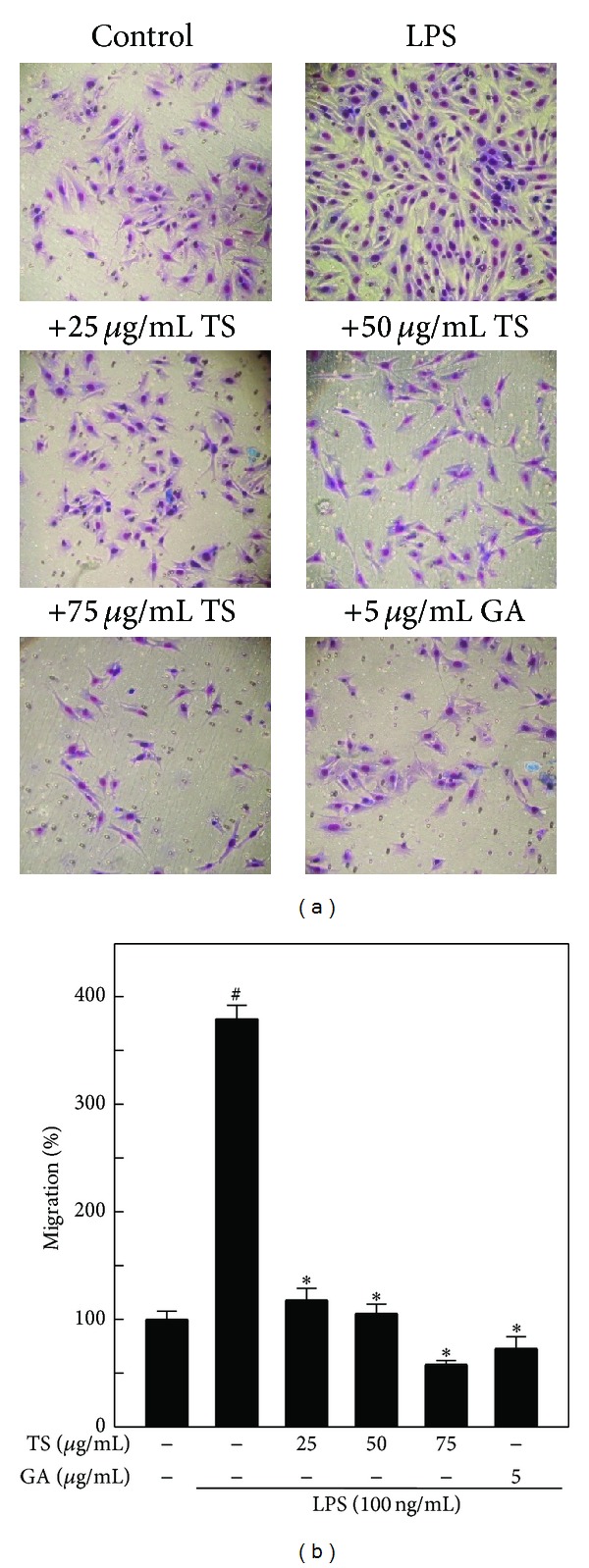
TS and GA inhibit the LPS-induced migration of A7r5 cells in the transwell assay. (a) Cells were preincubated with or without TS (25–75 *μ*g/mL) or GA (5 *μ*g/mL) for 2 h and then stimulated with LPS (100 ng/mL) for 24 h. Cells that migrated to the lower membrane were photographed (200x magnification). (b) The percentage of migrated cells was quantified and expressed relative to untreated cells (control), which represented 100%. To quantify migration, cells were counted in three microscopic fields per sample. The results are presented as the mean ± SD of three assays. ^#, ∗^indicates significant difference (*P* < 0.05) in comparison to control or LPS-treated groups, respectively.

Migration is a key step in the metastatic process. To establish the antimetastatic activity of TS and GA, we investigated their effect on the LPS-induced migration of A7r5 cells using a transwell migration chamber assay. As shown in Figure 5(a), LPS (100 ng/mL) treatment markedly increased A7r5 cell migration, as determined by the number of cells that migrated into the lower chamber. In contrast, preincubating A7r5 cells with TS (25–75 *μ*g/mL) and GA (5 *μ*g/mL) significantly inhibited LPS-induced migration after 24 h. Densitometric analyses revealed that LPS-induced A7r5 cell migration (380%) was reduced to 218%, 105%, 58%, and 72% following treatment with 25, 50, and 75 *μ*g/mL of TS or 5 *μ*g/mL of GA, respectively (Figure 5(b)).

### 3.7. TS and GA Downregulate LPS-Induced MMP-2, MPP-9, and t-PA Expression in A7r5 Cells

**Figure 6:**
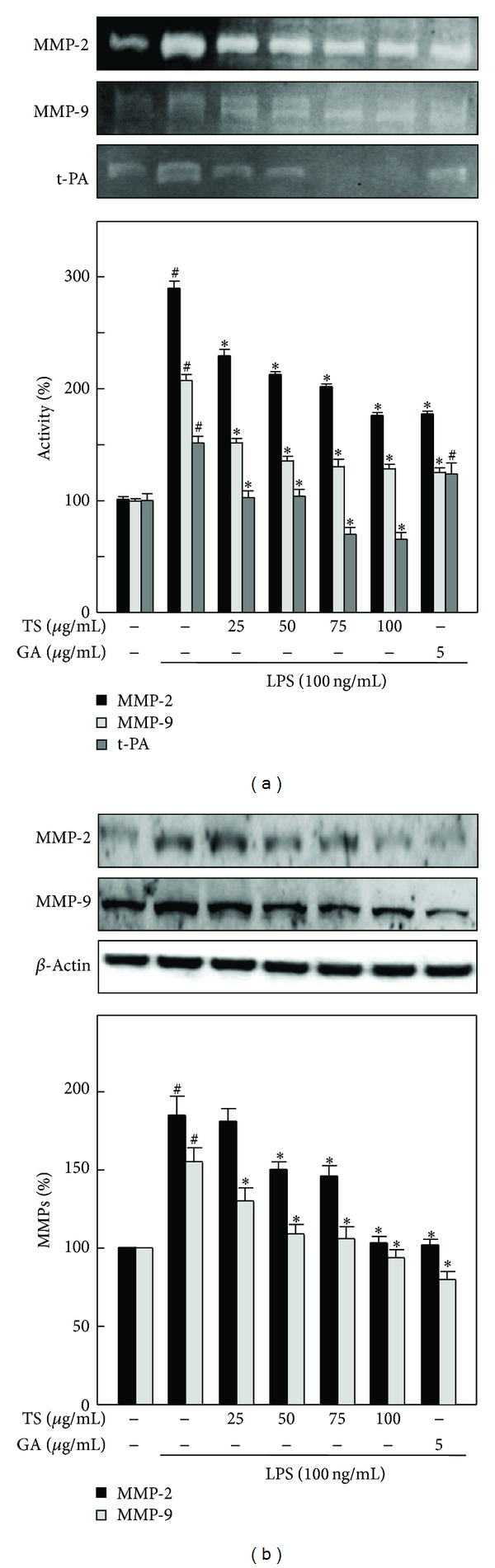
Inhibitory effects of TS and GA on LPS-induced MMP activity and expression in A7r5 cells. Cells were preincubated with or without TS (25–75 *μ*g/mL) or GA (5 *μ*g/mL) for 2 h and then stimulated with LPS (100 ng/mL) for 24 h for gelatin zymography and 12 h for western blotting. (a) Cell culture media was then subjected to gelatin zymography to analyze the activities of MMP-2, MMP-9, and t-PA. The activities of these proteins were subsequently quantified by densitometric analysis. (b) Western blotting was performed to analyze MMP-2 and MMP-9 protein levels. Proteins (50 *μ*g) from each sample were resolved by 8%–10% SDS-PAGE. *β*-Actin was used as an internal control. Relative changes in the protein bands were measured using AlpaEaseFc 4.0 software. Densitometric analysis, with the control being 100%, is shown below the gel data. The results represent the mean ± SD of three assays. ^#, ∗^indicates significant difference (*P* < 0.05) in comparison to control or LPS-treated groups, respectively.

The effects of TS and GA on LPS-induced A7r5 cells migration were further investigated by measuring the activities of MMPs and t-PA. As shown in Figure 6(a), intercellular MMP-2, MMP-9, and t-PA activities were detected in unstimulated cells by gelatin zymography, and the activities of MMP-2, MMP-9, and t-PA were markedly increased (290%, 210%, and 135%, resp.) after treatment with 100 ng/mL of LPS for 24 h. However, pretreatment with TS significantly inhibited the LPS-induced MMP-2, MMP-9, and t-PA activities in a dose-dependent manner. Treatment with GA (5 *μ*g/mL) also significantly inhibited the LPS-induced MMP-2 and MMP-9 activities, but not t-PA activity (Figure 6(a)). Consistent with the results of the gelatin zymography assay, LPS exposure greatly increased the protein expression levels of MMP-2 and MMP-9 to 190% and 170%, respectively, whereas cells pretreated with TS displayed a dose-dependent reduction in MMP-2 and MMP-9 protein expression following LPS stimulation for 12 h (Figure 6(b)). Pretreatment with GA also showed a significant reduction in LPS-induced MMP-2 and MMP-9 expression in A7r5 cells. Thus, it is possible that TS and GA can inhibit A7r5 cells migration by downregulating MMP-2 and MMP-9 expression.

### 3.8. TS and GA Inhibit LPS-Induced VEGF, PDGF, and VCAM-1 Expression in A7r5 Cells

**Figure 7:**
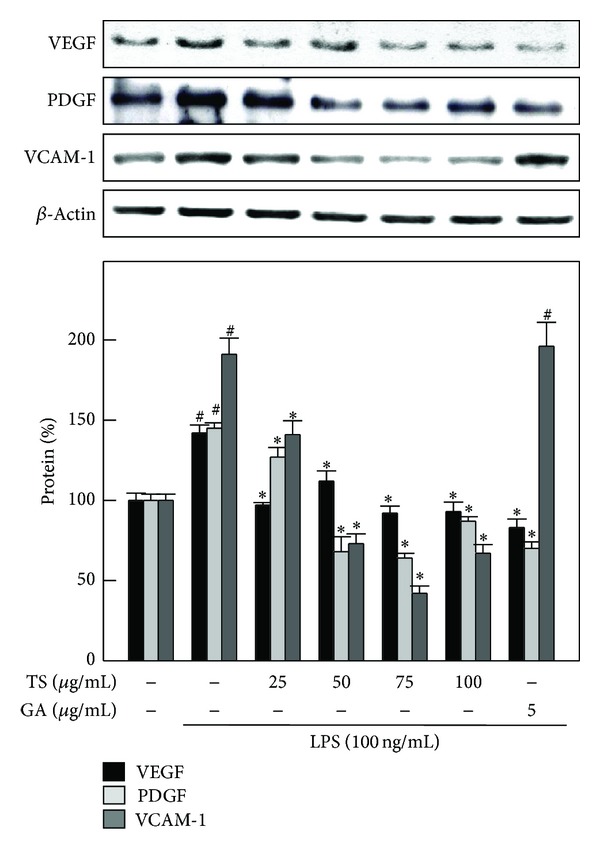
TS and GA mediatedownregulation of VEGF, PDGF, and VCAM-1 expression in LPS-activated A7r5 cells. Cells were preincubated with or without TS (25–100 *μ*g/mL) or GA (5 *μ*g/mL) for 2 h and were then stimulated with LPS (100 ng/mL) for 8 h. Proteins (50 *μ*g) from each sample were resolved by 8%–12% SDS-PAGE. *β*-Actin was used as a loading control. Relative changes in protein bands were measured using AlpaEaseFc 4.0 software. Densitometric analysis, with the control being 100%, is shown below the gel data. The results represent the mean ± SD of three assays. ^#, ∗^indicates significant difference (*P* < 0.05) in comparison to control or LPS-treated groups, respectively.

To determine whether TS and GA inhibit VEGF and PDGF expression, A7r5 cells were pre-incubated with TS (25–100 *μ*g/mL) and GA (5 *μ*g/mL), and VEGF and PDGF expression was stimulated by addition of LPS (100 ng/mL) for 12 h. Then, protein expression levels were assessed by western blot analyses. As shown in Figure 7, LPS treatment caused an approximately 140% increase in VEGF expression compared to unstimulated A7r5 cells (100%). However, pretreatment with TS and GA significantly reduced the LPS-induced increased in VEGF expression. In A7r5 cells, TS and GA pretreatment also caused a dose-dependent reduction in LPS-induced PDGF expression (Figure 7). Next, we examined the effects of TS and GA on LPS-induced VCAM-1 expression in A7r5 cells. LPS treatment caused approximately a 190% increase in VCAM-1 expression in A7r5 cells. Pretreatment with TS significantly inhibited the LPS-induced increase in VCAM-1 expression, whereas GA failed to inhibit LPS-induced VCAM-1 expression in A7r5 cells. This pronounced inhibition of VCAM-1 expression was observed at a concentration of 75 *μ*g/mL TS (Figure 7).

### 3.9. TS and GA Suppress LPS-Induced NF-*κ*B Activity by Inhibiting I-*κ*B*α* Degradation in A7r5 Cells

**Figure 8:**
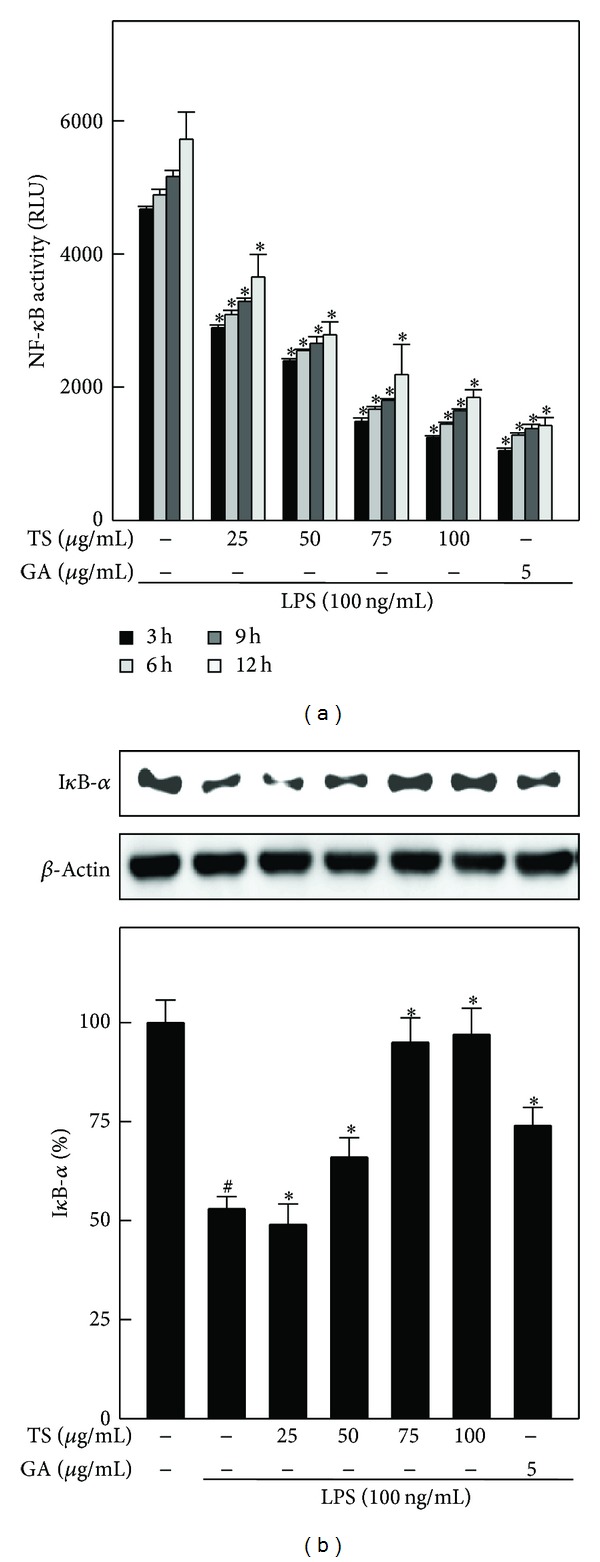
TS and GA suppress LPS-induced NF-*κ*B transcriptional activation and I*κ*B degradation in A7r5 cells. (a) A7r5 cells were transiently transfected with SEAP plasmids using lipofectamine. NF-*κ*B transcriptional activation was measured after preincubation with or without TS (25–100 *μ*g/mL) or GA (5 *μ*g/mL) for 2 h, followed by stimulation with LPS (100 ng/mL) for 3, 6, 9, and 12 h. Cell lysates were mixed with luciferase reagents and quantified using a illuminometer. Relative NF-*κ*B activity was calculated by dividing the relative luciferase unit (RLU) of treated cells by the RLU of untreated cells. (b) Western blotting was performed to analyze I*κ*B protein levels. Cells were preincubated with or without TS (25–100 *μ*g/mL) or GA (5 *μ*g/mL) for 2 h and then stimulated by LPS (100 ng/mL) for 45 min. *β*-Actin was used as an internal control. Relative changes in protein bands were measured using AlpaEaseFc 4.0 software. Densitometric analysis, with the control being 100%, is shown below the gel data. The results are presented as the mean ± SD of three assays. ^#, ∗^indicates significant difference (*P* < 0.05) in comparison to control or LPS-treated groups, respectively.

NF-*κ*B is an important transcription factor that mediates proinflammatory responses. Therefore, we sought to determine whether TS and GA could suppress LPS-induced NF-*κ*B activation in A7r5 cells. To monitor NF-*κ*B activity, NF-*κ*B-dependent transcription was measured with a luciferase reporter construct that was stably transfected into A7r5 cells. This luciferase reporter assay showed that LPS stimulation for 3–12 h caused a time-dependent increase in NF-*κ*B transcriptional activity, as determined by increased luciferase intensity. However, TS pretreatment significantly decreased NF-*κ*B transcriptional activity in a dose- and time-dependent manner (Figure 8(a)). A similar time-dependent effect was also observed after GA treatment. The nuclear translocation and transcriptional activation of NF-*κ*B are regulated by its inhibitor, I-*κ*B*α*. Therefore, we examined the effects of TS and GA on LPS-induced I-*κ*B*α* protein stability in A7r5 cells. As shown in Figure 8(b), I-*κ*B*α* was significantly degraded in response to treatment with LPS for 30 min, and this degradation was significantly inhibited upon pretreatment with TS or GA.

### 3.10. TS and GA Inhibit LPS-Induced MAPK Activation in A7r5 Cells

**Figure 9:**
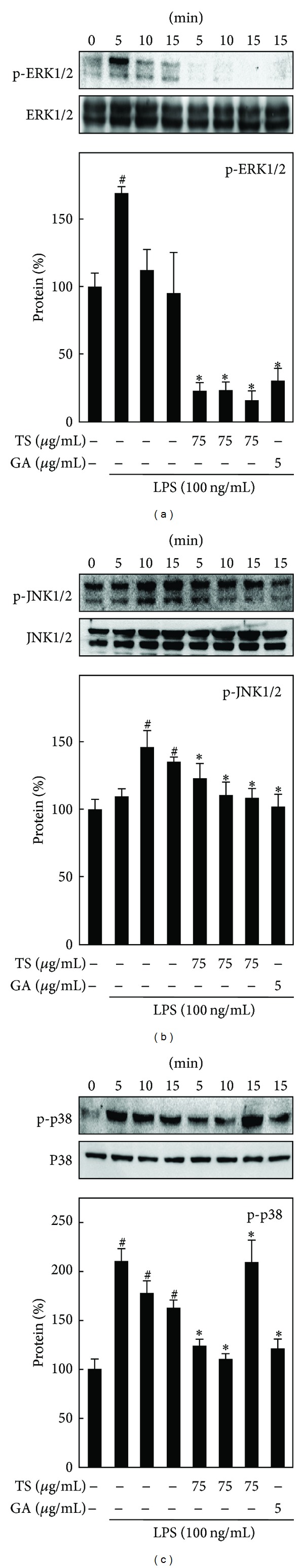
TS and GA attenuate MAPK signaling pathways in LPS-activated A7r5 cells. Cells were preincubated with or without TS (75 *μ*g/mL) or GA (5 *μ*g/mL) for 2 h and then stimulated by LPS (100 ng/mL) for 5–15 min. The phosphorylation of ERK1/2 (a), JNK1/2 (b), and p38 MAPK (c) was determined by western blot analysis with specific antibodies. Protein (50 *μ*g) from each sample was resolved by 10% SDS-PAGE, followed by western blotting. *β*-Actin was used as an internal control. Relative changes in protein bands were measured using AlpaEaseFc 4.0 software. Densitometric analysis, with the control being 100%, is shown just below the gel data. The results are presented as the mean ± SD of three assays. ^#, ∗^indicates significant difference (*P* < 0.05) in comparison to control or LPS-treated groups, respectively.

MAPKs, which are mainly composed of three subfamilies, ERK1/2, JNK1/2, and p38 MAPK, are serine/threonine protein kinases that are involved in the production of proinflammatory cytokines in response to various extracellular stimuli. To determine the inhibitory effects of TS and GA on LPS-induced MAPK activation in A7r5 cells, cells were preincubated with TS (75 *μ*g/mL) or GA (5 *μ*g/mL) and exposed to LPS (100 ng/mL) for 5–15 min. As shown in Figures 9(a), 9(b), and 9(c), LPS treatment caused a marked increase in the phosphorylation of ERK1/2, JNK1/2, and p38 MAPK proteins at 5, 15, and 5 min, respectively, whereas LPS-induced ERK1/2, JNK1/2, and p38 MAPK phosphorylation was significantly inhibited by TS and GA treatment (Figures 9(a), 9(b), and 9(c)). Importantly, there were no changes observed in the total protein levels of ERK1/2, JNK1/2, and p38 MAPK (Figures 9(a), 9(b), and 9(c)).

## 4. Discussion

*Toona sinensis* is a popular leafy vegetable in Taiwanese and Chinese cuisines that is also used as a traditional Chinese medicine. Previously, we demonstrated that aqueous leaf extracts from TS and their major bioactive compound, GA, inhibited LPS-induced inflammation in macrophage and mouse models [15]. Atherosclerosis involves inflammation and the migration of vascular SMCs. Pretreatment with TS and GA inhibited LPS-induced inflammation in cultured aortic smooth muscle (A7r5) cells by attenuating iNOS and COX-2 expression. Additionally, TS and GA treatment inhibited LPS-mediated SMC migration by suppressing MMPs, t-PA, VEGF, PDGF, and ICAM-1 expression. Moreover, TS- and GA-mediated inhibition of LPS-induced inflammation and migration in A7r5 cells was not attributable to cytotoxicity, as determined by MTT assay. Thus, to our knowledge, this study is the first to reveal the antiatherosclerotic action of this potentially beneficial medicinal plant.

Atherosclerosis is a complicated inflammatory process that can lead to vascular dysfunction. In A7r5 cells, inflammatory stimuli such as LPS, the major component of the Gram-negative bacterial cell wall, induces iNOS and COX-2 transcription and transduction, resulting in the production of proinflammatory cytokines and chemokines [25]. Therefore, A7r5 cells provide an excellent model for screening cardiovascular inflammation drugs and for subsequent evaluations of potential inhibitors on pathways that produce proinflammatory cytokines and chemokines [26]. Utilizing *in vitro* ELISA and western blot analyses, we found that pretreatment with TS (25–100 *μ*g/mL) and GA (5 *μ*g/mL) inhibited LPS-induced proinflammatory molecules, including NO and PGE_2_, in a dose-dependent manner. In particular, the profound inhibition of LPS-induced NO and PGE_2_ was observed at a concentration of 75 *μ*g/mL of TS. Furthermore, the expression of their precursors, iNOS and COX-2, was inhibited by TS and GA, which is consistent with our previous report that TS (25–100 *μ*g/mL) and GA (2.5–10 *μ*g/mL) significantly as well as dose-dependently inhibited the LPS-induced iNOS and COX-2 expression in murine macrophage RAW 264.7 cells, whereas treatment with TS (100 *μ*g/mL) and GA (10 *μ*g/mL) alone does not show any significant alterations in iNOS and COX-2 proteins [15]. In addition, our previous study also demonstrated that pretreatment of mice with TS (100 mg/kg b·w) and GA (5 mg/kg b·w) significantly inhibited the LPS-induced TNF-*α* and IL-1*β* release into the circulation [15]. Inhibition of pathological inflammation is a promising therapeutic strategy for many cardiovascular inflammatory diseases, including atherosclerosis and thrombosis [27]. Recently, increasing attention has been paid to identifying anti-inflammatory molecules from dietary sources and determining their underlying molecular mechanisms of action, with the rationale that a rationally designed nutraceutical supplement or drug could provide a safer and widely available source for preventing inflammation-related cardiovascular diseases [28].

SMC migration occurs during vascular injury, atherosclerosis, and vascular development. A number of studies have demonstrated that MMPs and PA are required for breaking down the extracellular matrix and allowing the migration of SMCs towards the subendothelial area [29, 30]. These observations highlight the possibility that MMPs and PA may act as important mediators of LPS-induced SMC migration. Therefore, the inhibition of MMP- and/or t-PA-mediated migration represents potential treatment for preventing or inhibiting SMC migration and atherosclerosis. Previous studies also demonstrated that LPS induces the transcription of MMP-2, MMP-9, and t-PA through the upregulation of NF-*κ*B and MAPKs [31, 32]. In this study, we demonstrated that treatment with TS (25–100 *μ*g/mL) significantly inhibited LPS-induced MMP-2, MMP-9, and t-PA activity or expression, whereas treatment with GA (5 *μ*g/mL) only inhibits MMP2 and MMP9, but not tPA activity in A7r5 cells.

Increased expression of adhesion molecules and angiogenic factors on the surface of A7r5 cells may play a key role in cardiovascular diseases [13]. LPS has been shown to be a critical inducer of adhesion molecules and angiogenic factors both *in vitro* and *in vivo *[31]. Vascular SMC migration begins with the stimulation of cell surface receptors, including VEGF and PDGF, which activate signal transduction pathways that promote the cytoskeletal remodeling, changes in the adhesiveness of SMCs to the matrix, and activation of motor proteins [33]. After percutaneous intervention, SMC proliferation and migration represent the end result of the natural healing processes triggered by vascular injury, whereas aberrant migration promotes the onset of atherosclerosis and cardiovascular diseases [33]. Leukocyte adhesion to arterial endothelial cells is thought to be an important step in the development of atherosclerosis [34]. It has been established that adhesion molecules are strong predictors of atherosclerotic lesion development and the progression of cardiovascular events [34]. These include both secreted molecules, such as the chemokine family of chemoattractant cytokines, and surface-expressed cell adhesion molecules, including the selectin and immunoglobulin families [35]. In our previous study, we reported that treatment with TS (25–75 *μ*g/mL) significantly as well as dose-dependently inhibited the VEGF expression in human premyelocytic leukemia HL-60 cells [16]. In this study we demonstrate that TS (25–100 *μ*g/mL) and GA (5 *μ*g/mL) significantly diminish the expression of VEGF, PDGF, and VCAM-1 in LPS-challenged SMCs, the pronounced inhibition was observed at a concentration of 75 *μ*g/mL of TS, whereas GA (5 *μ*g/mL) inhibits LPS-induced VEGF and PDGF expression, but not VCAM-1 expression in A7r5 cells. Thus, TS and GA play a major role in the prevention of SMC migration and atherosclerosis.

ROS play a key role in promoting SMC proliferation and inflammation in the vessel wall, which are features that stimulate atherosclerosis [36]. In our study, LPS treatment markedly increased ROS generation in SMCs, and TS and GA significantly inhibited the generation of ROS. In the vasculature, several enzymes induce ROS generation, including NADPH oxidase [37]. The phagocytic NADPH oxidase generates superoxide through the assembly of multisubunit protein complexes, such as the membrane bound proteins, gp91phox and p22^phox^, and the cytosolic component, p47^phox^ [38]. During oxidase activation, p47^phox^ is phosphorylated and translocates to the membrane, leading to vascular adventitia [37, 39]. Our investigation demonstrates that TS (75 *μ*g/mL) and GA (5 *μ*g/mL) significantly reduced LPS-induced intracellular ROS accumulation and p47^phox^ expression. Therefore, TS and GA represent potential therapeutics for the prevention of atherosclerosis.

NF-*κ*B, a Rel-family transcription factor, is known to play critical roles in the regulation of genes involved in cell proliferation/migration and the coordination of the expression of proinflammatory mediators, including iNOS, COX-2, TNF-*α*, IL-1*β*, and IL-6 [4]. The fact that NF-*κ*B activation is inhibited to various degrees by different antioxidants strongly suggests that ROS generation might play an important role in regulating these redox-sensitive transcriptional pathways in atherogenesis. NF-*κ*B has two levels of redox regulation: one in the cytoplasm and another in the nucleus. The former involves the phosphorylation of two serine residues (S32 and S36) on I*κ*B*α*, which results in its polyubiquitination and subsequent degradation by the 26S proteasome, permitting the unmasking of the nuclear localization signal and translocation of activated NF-*κ*B into the nucleus. The latter process involves the direct redox modification of specific cysteine residues in the DNA-binding domain of nuclear NF-*κ*B [4]. Using a luciferase reporter assay, we found that LPS-induced NF-*κ*B transcriptional activity was inhibited by TS (25–75 *μ*g/mL) and GA (5 *μ*g/mL) in a dose- and time-dependent manner. Our previous study also partially supports this notion that LPS-induced NF-*κ*B activation in mice was significantly prevented by TS (100 mg/kg b·w) and GA (5 mg/kg b·w), which was assessed by bioluminescence imaging [15]. In addition, the nuclear translocation and transcriptional activation of NF-*κ*B is tightly regulated by an inhibitor subunit known as I-*κ*B*α*. Upon stimulation, cytoplasmic I-*κ*B*α* is phosphorylated and subsequently degraded by the proteasome, which allows NF-*κ*B to translocate to the nucleus, where it activates NF-*κ*B-responsive genes. TS and GA were found to prevent LPS-induced degradation of I-*κ*B*α*, which is associated with the suppression of NF-*κ*B activity in LPS-induced A7r5 cells. Generally, inhibitors of NF-*κ*B activation have been shown to suppress SMC migration through the inhibition of MMPs and t-PA activity [40–42]. Suppressing NF-*κ*B activity might also block SMC migration and other factors that bind to these regulatory elements; therefore, NF-*κ*B inhibition represents a promising approach to inhibit the synthesis of MMPs and t-PA. Our findings suggest that TS- or GA-mediated inhibition of SMC migration may be mediated, in part, through the suppression of MMP-2, MMP-9, and t-PA expression in response to modulation of the NF-*κ*B signaling pathway.

The presence of exogenous ROS can activate MAPK, which is upstream of the NF-*κ*B pathway, which is important for inflammation and cell proliferation/migration [43]. Furthermore, the LPS-induced generation of ROS and activation of MAPKs, including JNK and p38 MAPK, stimulate proinflammatory cytokine production, while activation of ERK is required for the expression of adhesion molecules in vascular SMC [29, 30, 32, 44]. In this study, we found that incubation of A7r5 cells with LPS activated all three of these MAPKs, which is consistent with a previous report that showed that LPS treatment induced MAPK phosphorylation in human aortic SMC [31]. Importantly, pretreatment with TS or GA inhibited the LPS-induced activation of ERK1/2, JNK1/2, and p38MAPK. These results indicate that TS- and GA-mediated inhibition of iNOS and COX-2 expression is associated with the suppression of MAPKs. The ROS-MAPK pathway has been implicated in the regulation of SMC proliferation and migration [12], as several studies in different cell types have indicated that MAPKs play a central role in regulating the expression of MMPs and t-PA [45, 46]. Therefore, inhibition of the MAPK pathway might be a useful strategy to prevent SMC migration. Our data demonstrate that TS and GA inhibit the phosphorylation of ERK1/2, JNK1/2, and p38MAPK, resulting in a concurrent reduction in the levels of MMPs and t-PA, and suggesting a possible mechanism for the inhibition of MMPs or t-PA synthesis and SMC migration by TS or GA. These findings suggest that MAPKs could represent a link between ROS, SMC inflammation, and migration in atherosclerosis.

Plant secondary metabolites have received particular attention due to new findings in regard to their biological activities. In particular, flavonoids, triterpenes, tannins, chalcones, and polyphenols from plant sources possess various pharmacological properties, including antioxidant, anti-inflammatory, antimutagenic, and anticancer effects [47]. Previously, we isolated nine major compounds, including gallic acid (GA), methyl gallate, ethyl gallate, kaempferol, kaempferol 3-*O*-*β*-D-glucoside, quercetin, quercitrin, quercetin 3-*O*-*β*-D-glucoside, and rutin, from aqueous leaf extracts of *Toona sinensis *[14, 23]. The total phenolic content of TS was estimated to be 130 mg ± 26 mg gallic acid (pyrocatechol), equivalents/g of plant extracts [23]. The yield of GA, the natural phenolic component purified from TS, was approximately 10% [23]. Although it remains unclear which of the components of TS are active compounds, the biological activities of polyphenols have gained much attention in recent years, especially the antioxidant activity of GA [48].

GA is widely distributed in various plants and fruits. Although the therapeutic utility of GA is unknown, it is a common presence in fruits and food and its small molecular weight (170 Da) might be an advantageous in terms of its safety and dosing design. Utilizing a bioassay-guided fraction, we found that GA possessed potent antioxidant and anticancer effects [23]. Kroes et al. [48] reported that GA inhibited zymosan-induced paw edema in mice, and subsequent *in vitro* studies revealed that this inhibitory effect was due to functional modulation of polymorphonuclear leucocytes. GA also showed potent antioxidant activity via scavenging superoxide anions, and it inhibited NADPH-oxidase activity and myeloperoxidase release. An *in vitro* study showed that GA inhibited intracellular ROS accumulation and lipid peroxidation in Kainic acid-induced PC12 cells by downregulating MAPK signaling pathways [49]. GA inhibits LPS-induced NF-*κ*B signaling by inhibiting RelA acetylation in A549 lung cancer cells [50]. Our previous study also revealed that GA inhibits iNOS and COX-2 expression by downregulating NF-*κ*B activity both *in vitro* and *in vivo* [15]. These results imply that GA is one of the active compounds responsible for the inhibition of LPS-induced SMC inflammation and migration. Further bioassay-directed fractionations leading to the identification and purification of other compounds responsible for the antiatherosclerotic effects of TS are warranted.

There are several herbal products including St. John's wort, motherwort, *Gingko biloba* garlic, grapefruit juice, hawthorn, saw palmetto, danshen, echinacea, tetrandrine, aconite, yohimbine, gynura, licorice, and black cohosh which have direct effects on the cardiovascular or hemostatic system, whereas few have indirect effects through interactions with medications that could lead to serious consequences [51]. Previous studies with rodent model demonstrate that administration of TS does not show any possible lethal effects at concentrations as high as 1 g/kg body weight [19, 20]. However, further studies regarding its pharmacokinetics, pharmacodynamics, drug efficacy, and side effects are highly warranted. Moreover, the herb-drug interactions are especially relevant when cardiovascular medications with a narrow therapeutic index, such as digoxia and warfarin, are coadministered with herbs that can potentiate or reduce pharmacologic effect. Therefore, in the near future we aimed to investigate the potential effects of TS with cardiovascular drugs.

## 5. Conclusions

In conclusion, TS and GA inhibit LPS-induced inflammation in cultured aortic smooth muscle cells *via* attenuation of iNOS/COX-2 expression. Moreover, TS and GA suppress LPS-induced SMC migration through the inhibition of MMPs, t-PA, growth factors, and adhesion molecules. We also provide a feasible mechanism by which TS- and GA-mediated inhibition of the NF-*κ*B and/or MAPK signaling pathways plays a major role in their anti-inflammatory and antimigratory effects. These results indicate that *Toona sinensis* and its major bioactive compound, gallic acid, have potent anti-inflammation and antimigration activities that contribute to their atherosclerosis chemopreventive potential. Therefore, *Toona sinensis* could be used as an accessible source of natural antioxidants and possible nutraceutical supplements, with potential applications in the pharmaceutical industry.

## Abbreviations

TS: *Toona sinensis*
GA: Gallic acid
LPS: Lipopolysaccharide
SMC: Smooth muscle cells
ROS: Reactive oxygen species
NO: Nitric oxide
iNOS: Inducible nitric oxide synthase
PGE_2_: Prostaglandin E_2_
COX-2: Cyclooxygenase-2
MMP: Matrix metalloproteinase
t-PA: Tissue-type plasminogen activator
VEGF: Vascular endothelial growth factor
PDGF: Platelet-derived growth factor
VCAM-1: Vascular cell adhesion molecule-1
NF-*κ*B: Nuclear factor-kappa B
I*κ*B: Inhibitor kappa B
MAPK: Mitogen activated protein kinase
JNK1/2: c-JUN N-terminal kinase1/2
ERK1/2: Extracellular signal-regulated kinase1/2.
